# Utility of I-*Sce*I and *CCR5*-ZFN nucleases in excising selectable marker genes from transgenic plants

**DOI:** 10.1186/s13104-019-4304-2

**Published:** 2019-05-14

**Authors:** Bhuvan P. Pathak, Eliott Pruett, Huazhong Guan, Vibha Srivastava

**Affiliations:** 1 Dept. of Crop, Soil & Environmental Sciences, University of Arkansas, Fayetteville, AR USA; 2 Cell and Molecular Biology Program, University of Arkansas, Fayetteville, AR USA; 3 Fujian Provincial Key Laboratory of Crop Breeding, Fujian Agricultural & Forestry University, Fuzhou, China; 4 Dept. of Horticulture, University of Arkansas, Fayetteville, AR USA

**Keywords:** Meganuclease, I-*Sce*I, Zinc finger nuclease, Targeted excision, Genetic engineering

## Abstract

**Objectives:**

Removal of selection marker genes from transgenic plants is highly desirable for their regulatory approval and public acceptance. This study evaluated the use of two nucleases, the yeast homing endonuclease, I-*Sce*I, and the designed zinc finger nuclease, *CCR5*-ZFN, in excising marker genes from plants using rice and *Arabidopsis* as the models.

**Results:**

In an in vitro culture assay, both nucleases were effective in precisely excising the DNA fragments marked by the nuclease target sites. However, rice cultures were found to be refractory to transformation with the I-*Sce*I and *CCR5*-ZFN overexpressing constructs. The inducible I-*Sce*I expression was also problematic in rice as the progeny of the transgenic lines expressing the heat-inducible I-*Sce*I did not inherit the functional gene. On the other hand, heat-inducible I-*Sce*I expression in *Arabidopsis* was effective in creating somatic excisions in transgenic plants but ineffective in generating heritable excisions. The inducible expression of *CCR5*-ZFN in rice, although transmitted stably to the progeny, appeared ineffective in creating detectable excisions. Therefore, toxicity of these nucleases in plant cells poses major bottleneck in their application in plant biotechnology, which could be avoided by expressing them transiently in cultures in vitro.

**Electronic supplementary material:**

The online version of this article (10.1186/s13104-019-4304-2) contains supplementary material, which is available to authorized users.

## Introduction

Selection marker genes are indispensable tools in genetic engineering. Their presence in transgenic crops, however, could be detrimental [1], requiring methods for removing them from the plant. The most desirable outcome is to precisely delete the marker genes without creating off-target mutations. The Cre-*lox* site-specific recombination system is highly successful in achieving that goal [2–4], but it leaves a reactive footprint, the functional *lox* site, in the genome, rendering it non-reusable for the next round of transformation [5, 6].

The double-stranded break (DSB) repair mechanism has long been proposed as an alternative approach for excising marker genes, which can be repeatedly used in the same transgenic line as this mechanism destroys the target site by creating insertion–deletions (indels). Several nucleases, including meganucleases, ZFN, and CRISPR/Cas have been used for creating concomitant DSBs to achieve transgene deletions in the plant cells [7–11]. However, their applications in generating marker-free plants needs more investigation. This study evaluated the effectiveness of codon-optimized I-*Sce*I [12] and *CCR5*-ZFN [13] in excising genes in rice and *Arabidopsis* using overexpression and inducible expression approaches. These two nucleases were chosen because they have been successfully used in plant genome engineering [10, 14–16].

In this study, the expression of I-*Sce*I and *CCR5*-ZFN appeared to be deleterious as indicated by the failure to transform rice with the overexpression constructs, indicating their activity on non-canonical target sites. The inducible expression was ineffective in creating excisions in plants and/or transmitting them to the progeny. Retransformation approach, on the other hand, was successful in creating targeted excision in cultures in vitro. Therefore, the use of nucleases in plants is hampered by their genotoxic property and lower efficiencies, but retransformation of in vitro cultures could serve as a practical solution for creating targeted excisions, which could then be regenerated into plants. However, several ‘excision events’ will have to be screened for precise targeted excisions and the potential off-target mutations.

## Main text

### Methods

#### DNA constructs, plant transformation, and treatments

All constructs were prepared using the standard molecular biology techniques. The synthetic coding sequences of I-*Sce*I and *CCR5*-ZFN were provided by Drs. Holger Puchta (Karlsruhe, Germany) and Joseph Petolino (Dow Agro Sciences, Inc.), respectively. *Agrobacterium*-mediated and biolistics-mediated rice (Nipponbare) transformations have been described earlier [9, 17]. *Arabidopsis* (Col-0) transformation was done using the floral-dip method [18]. Heat-shock treatments of rice in vitro cultures, cut leaves or the seedlings was done by placing the tissues in the petri-dish or wrapped in aluminum foil in an incubator maintained at 42 °C for 3 h, followed by 72 h of recovery before scarifying the tissue for DNA/RNA isolation. For *Arabidopsis*, seedlings in the germination media (MS media without sucrose) were placed in 40 °C for 3 h followed by 48 h of recovery.

#### Molecular analysis

The PCR primers were designed using Primer Blast tool and verified in the IDT oligo-analyzer for the hairpin, self and heterodimer structures. They were also checked by BLAST to look for any potential non-specific sites in the rice and *Arabidopsis* genomes. Primers used in the present study are given in Additional file 1: Table S1. PCR was performed at 94 °C for 4 min followed by 40 cycles of 1 min at 58–60 °C and 1–2 min at 72 °C depending on the amplicon size (unless otherwise stated) using Emerald Amp PCR master mix (TaKaRa Inc.). All the PCR assays included the non-transformed rice or *Arabidopsis* genomic DNA as the negative control to screen for any non-specific amplification. For gene expression analysis, total RNA isolated using RNaesy kit (Qiagen Inc.) was subjected to real-time PCR using Super Script III one step qRT-PCR kit (Invitrogen) using manufacturer’s instructions. Relative expression was calculated against wild-type using 2^ΔΔCt^ method [19], and the Ct values were normalized against internal control, *Ubiquitin* or *Phytoene desaturase* genes. The purified PCR products were sequenced at Eurofin Genomics USA. Genomic DNA of selected lines were also analyzed on Southern blot using P32-labeled DNA probes.

### Results

#### Expression of I-*Sce*I and ZFN in rice

**Fig. 1:**
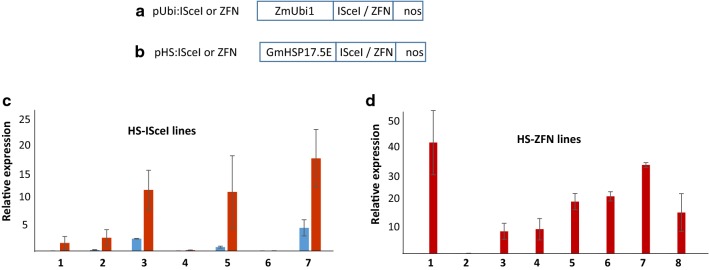
Expression of I-*Sce*I and ZFN in rice. **a**, **b** Overexpression and inducible constructs of I-*Sce*I or ZFN contain ZmUbi1 for constitutive overexpression or GmHSP17.5E for HS-inducible expression with *nos 3*′ as transcription termination sequence. **c**, **d** Real-time quantitative PCR analysis on total RNA isolated from the rice lines expressing HS inducible I-*Sce*I or ZFN gene. Relative expression against wild-type control is shown for each line. Bars show mean of two treatments with standard errors. Red and blue bars represent HS and room temperature (RT) samples, respectively. Note that ZFN expression at RT was close to the wild-type controls

The overexpression constructs consisting of ZmUbi1 promoter for I-*Sce*I or ZFN expression (Fig. 1a) were co-bombarded with hygromycin resistance gene (hygR) on the scutellar callus of rice cv. Nipponbare. The hygR gene consisted of hygromycin phosphotransferase gene driven by CaMV 35S promoter. No selectable clones were obtained with I-*Sce*I overexpression construct in two different experiments, suggesting geno-toxicity of I-*Sce*I in rice. With ZFN overexpression construct, 11 hygR lines were generated that were PCR-positive for ZFN gene. However, only 3 of these set a low number of seeds (10–30 seeds/line), indicating high rate of sterility in ZFN rice plants. The PCR analysis of the T1 plants from these three lines revealed lack of inheritance of the ZFN gene (Additional file 2: Figure S1). Therefore, strong expression of ZFN also generated toxicity in rice cells that severely hampered inheritance of the ZFN gene. The BLASTn analysis, (using default parameters—input: 33 or 18 bp; e-value threshold: 10; match/mismatch score: 1, − 3; gapopen: − 5 and gapextend: − 3) of 18 bp I-*Sce*I and 33 bp *CCR5* sites did not reveal match in the rice or *Arabidopsis* genome. The online tools for predicting off-target of I-*Sce*I are lacking, but five I-*Sce*I like sites [20] were also used in the BLASTn analysis, none of which found a 100% match in the rice or *Arabidopsis* genome. Off-target prediction of the *CCR5*-ZFN by Prognos tool [21] found 12 highly probable sites in the rice genome.

Next, inducible expression constructs consisting of GmHSP17.5E gene promoter expressing I-*Sce*I or ZFN (Fig. 1b) were co-transformed with hygR gene into Nipponbare callus. Seven I-*Sce*I and 8 ZFN lines were recovered, indicating curbed toxicity of the inducible I-*Sce*I and ZFN in rice. Expression analysis was conducted on heat-shock-treated (HS) cut leaves obtained from the greenhouse grown plants. Five HS–I*Sce*I lines and seven HS–ZFN lines showed several fold increase in the expression with respect to the untreated control, confirming proper regulation of these nucleases in the rice plant (Fig. 1c, d). The HS–ZFN lines showed normal growth and fertility, and transmitted ZFN activity to the progeny. The HS–I*Sce*I lines, on the other hand, did not transmit I-*Sce*I gene to the progeny and showed poor growth and high sterility, indicating toxicity of the basal expression of the inducible I-*Sce*I gene to the somatic and germ cells.

#### Characterization of inducible ZFN activity in excising marker gene in rice plants

**Fig. 2:**
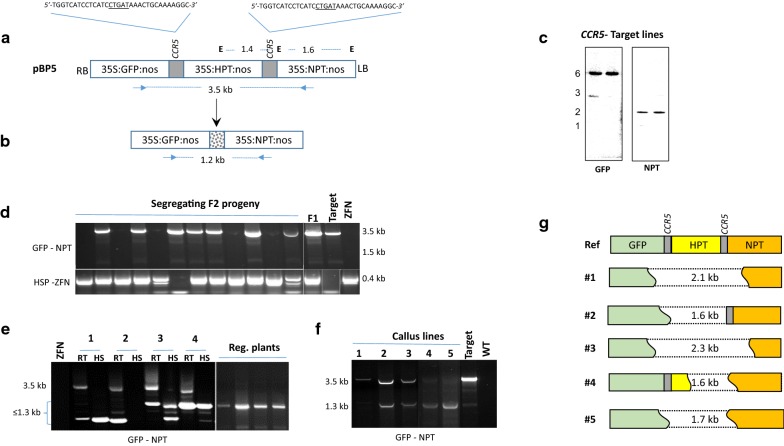
Characterization of HSP–ZFN in rice. **a** The *CCR5*-target construct in pPZP200 binary vector contains *GFP*, *HPT* and *NPT* genes. Each of which is controlled by *35S* promoter and *nos 3′* terminator. The *HPT* gene is flanked by 33 bp *CCR5* sequences (gray bars). Location of *Eco*R1 (E) sites and the fragment sizes are shown. **b** Predicted structure of ZFN-induced precise excision of *HPT* cassette with indels in between (dotted bar). PCR primer positions and predicted fragment sizes (in kb) are shown below each structure. **c** Southern blot analysis of rice lines transformed with pBP5. Genomic DNA was cut with *Eco*RI and hybridized with P^32^ labeled *GFP* or *NPT* probes. Fragment sizes are given in kb. **d** PCR analysis using primers located in *CCR5*-target sites (*GFP*–*NPT*) or ZFN gene (HSP–ZFN) on genomic DNA isolated from F2 plants derived from crosses between *CCR5*-target lines and HSP–ZFN lines. F1 parent, and *CCR5*-target and ZFN lines are also shown. **e** PCR across *CCR5* sites in the retransformed callus clones and the regenerated plants obtained by retransformation of HS–ZFN line #7 (Fig. 1d) with pBP5. The room temperature (RT) or heat-shocked (HS) samples of the selected calli clones (1–4) are shown with the regenerated plants obtained from them. ZFN line #7 serves as the negative control. **f** PCR across *CCR5* sites in the retransformed clones derived from the retransformation of *CCR5*-target lines with pHSP:ZFN construct. Target line and wild-type (WT) are included as controls. **g** Depiction of indels created by targeting of the two *CCR5* sites in the target site as determined by aligning the DNA sequences of selected ≤ 1.3 kb bands with pBP5 reference. Deletions sizes are given in each diagram

While the experiments with HS–I*Sce*I had to be discontinued due to problematic heritability of I-*Sce*I gene, HS–ZFN lines were cross-pollinated with *CCR5* target lines developed by transformation of Nipponbare rice with pBP5 that contains three gene cassettes, *GFP*, *HPT* and *NPT*, with a pair of 33 bp *CCR5* sites flanking the *HPT* cassette (Fig. 2a). Targeting of *CCR5* sites by ZFN could lead to the excision of *HPT* and fusion of the distal ends creating indels at the targeted sites (Fig. 2b). Five healthy F1 plants representing three different ZFN lines (lines #3, #6, #7; Fig. 1b) and two different *CCR5*-target lines (Fig. 2c) were heat-shocked and grown to maturity in the greenhouse. All F1 plants expressed *GFP* and the HS-induced ZFN activity, confirming the presence of *CCR5* target and ZFN constructs; however, excision of the *HPT* cassette was undetectable by PCR across *CCR5* sites (data not shown). Several F2 seedlings that were positive for *GFP* and ZFN were also heat-shocked and sacrificed for DNA isolation, but none showed the excision site (≤ 1.3 kb) in the PCR, while the presence of intact target site (3.5 kb) was evident in a number of them (Fig. 2d). Hence, HS-induced ZFN activity appeared suboptimal in creating detectable excisions in rice. This observation corroborates with that of Lu et al. [22], who reported low frequency targeting by heat-inducible ZFN in poplar.

#### Targeted excisions by retransformation

The failure in scoring targeted excisions in the F1 hybrids and their progeny derived from the crosses between HS–ZFN and *CCR5*-target lines raised questions whether ZFN expression was sufficient and the target locus was accessible to ZFN activity. To address these questions, reciprocal transformations were done, i.e., transformation of ZFN-expressing line with pBP5, and transformation of *CCR5*-target lines with pHS:ZFN. Retransformation of HS–ZFN line #7 with pBP5 generated 19 geneticin-resistant calli events that expressed *GFP*, indicating stable integration of the target construct in the genome. PCR across *CCR5* sites found that 17 of these lines showed both full-length *HPT* cassette (3.5 kb) and the excision site (≤ 1.3 kb) in the room temperature (RT) samples, 4 of which showed strong presence of excision site in the heat-shock (HS) samples (Fig. 2e). These data suggest that basal ZFN activity from HS:ZFN gene could induce targeting at *CCR5* sites but the targeting efficiency increased upon HS treatment. Four regenerated plants were obtained from these callus lines that also showed the ~ 1.3 kb excision site (Fig. 2e). Similarly, transformation of the *CCR5*-target lines with pHS:ZFN vector, produced 9 calli events, 4 of which showed ~ 1.3 kb excision band in HS-treated calli (Fig. 2f). Sequencing of five excision sites (≤ 1.3 kb) from these experiments found complete or partial excision of HPT cassette with large indels (> 1.5 kb) spreading into the adjacent sequences (Fig. 2g). In summary, HS-induced ZFN activity is capable of creating targeted excisions in rice cultures in vitro.

#### Inducible I-*Sce*I mediated marker excision in *Arabidopsis*

**Fig. 3:**
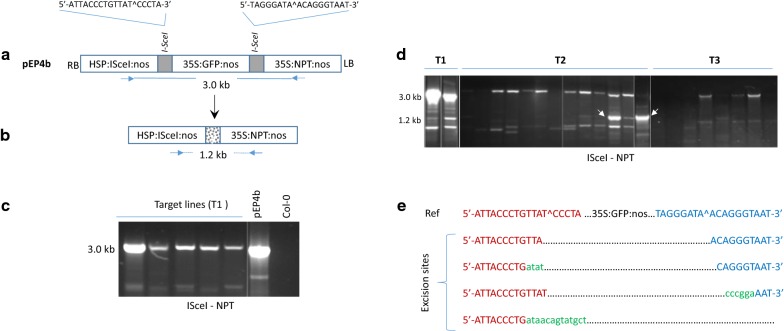
Characterization of HS-inducible I-*Sce*I in *Arabidopsis*. **a** I-*Sce*I target construct, pEP4b, in pPZP200 binary vector contains HS-inducible I-*Sce*I, *GFP*, and *NPT* expression units with 18 bp I-*Sce*I target sites (gray bars) flanking the *GFP* cassette. **b** Predicted structure of the target site upon precise excision of *GFP* cassette with indels at the targeted site (dotted bar). PCR primer positions and the fragment sizes are shown by blue arrows. **c** PCR analysis of the first generation transgenic (T1) lines using primers located in I-*Sce*I and *NPT* cassettes with pEP4b and wild-type Col-0 as controls. **d** PCR analysis of three generations: T1 parents, T2, and T3 progeny to detect excision of *GFP* cassette. White arrows indicate bands that were purified and subjected to Sanger sequencing. **e** DNA sequences of ~ 1.2 kb predicted excision bands were aligned with the pEP4b reference to determine indels at the targeted sites. Red and blue fonts represent the two I-*Sce*I sites with predicted breakpoints (^). Dotted lines indicate deletions and green small letters show insertions

Since I-*Sce*I expression was highly toxic in rice, further experiments with inducible I-*Sce*I were carried out in *Arabidopsis*. For this purpose, pEP4b construct was developed that contains a pair of I-*Sce*I target sites flanking the *GFP* cassette, the kanamycin resistance (*NPT*) cassette, and the HS-inducible I-*Sce*I expression cassette (Fig. 3a). The excision of the *GFP* cassette in this construct would result in fusion of I-*Sce*I and *NPT* cassette with indels in between (Fig. 3b). Transformation of *Arabidopsis* Col-0 with pEP4b generated 11 kanamycin resistant T1 lines that contained a full-length integration of pEP4b construct in the PCR assay (Fig. 3c). Fertility in these T1 plants was substantially low, indicating I-*Sce*I toxicity in the germline (≤ 10× lower compared to that of the healthy *Arabidopsis* plants). Germination of T2 seedlings on kanamycin-containing (50 mg/l) media displayed gradual lethality and receding GFP expression in all lines; however, seedlings could be rescued on a kanamycin-free medium and grown to maturity. This indicates that large indels possibly occurred at the target sites, eliminating *NPT* and *GFP* activity. The rescued T2 seedlings were analyzed by PCR to determine the target and excision sites, indicated by 3.0 and 1.2 kb products, respectively (Fig. 3a, b). The majority of T2 progeny either failed to show these PCR products or showed their weak presence, indicating large indels at the target site in the majority of the tissue. Two T2 lines showed strong presence of ~ 1.2 kb band (Fig. 3d: white arrows), which was sequenced and found to contain the near-precise excision of GFP cassette with very small indels at the target sites (Fig. 3e). The analysis of T3 seedlings, however, suggested that the observed excision site in the T2 parents was not transmitted to the progeny as none showed the 1.2 kb band (Fig. 3d). In summary, HS–I*Sce*I was able to generate targeted excisions in the *Arabidopsis* seedlings, but inheritance of the excision site was questionable.

### Conclusions

Potential genotoxicity of I-*Sce*I and *CCR5*-ZFN appears to be a major bottleneck in their application in plant biotechnology. However, retransformation of in vitro cultures could be used as an effective approach for excising of marker genes and regenerating the marker-free plants.

## Limitations

The main limitation of this study is that rice and *Arabidopsis* genomes could contain off-target sites of I-*Sce*I and *CCR5*-ZFN nucleases that would prohibit the application of these nucleases in these plant species. A larger set of nucleases, e.g., newly designed ZFNs or TALENs should be tested to determine if other nucleases can be used successfully in achieving marker excision in these plant species.

## Additional files

**Additional file 1: Table S1.** Primers used in this study.

**Additional file 2: Figure S1.** Molecular analysis of rice lines transformed with ZFN overexpression construct. (**a**) ZFN overexpression construct containing maize Ubiquitin-1 (ZmUbi) promoter, ZFN coding region and nopaline synthase (nos) 3’ transcription terminator. Primer positions and their product size are shown. (**b**) PCR analysis of 13 primary transgenic plants (T0) representing 11 transgenic events. (**c**) PCR analysis of T1 progeny from three T0 plants # 1, 2-1 and 3. **d**, **e** PCR analysis of additional T1 progeny from line #3. Product sizes are shown. Arrows indicate expected products in each gel. The PCR conditions for Figures (b–d) are mentioned in the main text. The PCR for 0.09 kb product (Figure e) was performed at 95 °C for 3 min followed by 30 cycles of 95 °C for 30 s, 60 °C for 30 s, and 72 °C for 30 s.

## Data Availability

The vectors generated in this study can be requested from the corresponding author. All data generated and analyzed during this study are included in this published article and its additional information.

## Abbreviations

Indels: insertions–deletions
ZFN: zinc finger nuclease
GFP: green fluorescent protein
HPT: hygromycin phosphotransferase
NPT: neomycin phosphotransferase
GmHSP17.5E: glycine max heat-shock protein 17.5E
ZmUbi1: Zea mays ubiquitin 1
35S: cauliflower mosaic virus 35S RNA gene promoter
nos 3′: agrobacterium tumefaciens nopaline synthase 3′ transcription termination sequence
HS: heat-shock
CCR5: C–C motif chemokine receptor 5 (from human genome)
PCR: polymerase chain reaction
