# Supplementary material for: Utility of I-SceI and CCR5-ZFN nucleases in excising selectable marker genes from transgenic plants
Source: BMC Res Notes. 2019 May 14;12:272. doi: 10.1186/s13104-019-4304-2 (PMC6518718; doi:10.1186/s13104-019-4304-2)
Supplement: Supplementary file 1 — Additional file 1: Table S1. Primers used in this study. [file 13104_2019_4304_MOESM1_ESM.docx]

**Additional File S1:**

**Table S1: List of the primers used in this study**

| **Primers** | **Sequence (5’ – 3’)** | **Application** |
| --- | --- | --- |
| pEP4b primers | TTCTCCACACCATGTACGCA | Genotyping Arabidopsis pEP4b lines |
|  | GCATCGCCTTCTATCGCCTT |  |
| pBP5 primers | AAGACCCCAACGAGAAGC | Genotyping rice pBP5 lines |
|  | CTCGATGCGATGTTTCGCTT |  |
| pHSP:ZFN primers | CCTTGCGTACATGGTGTGGA | Genotyping HS-ZFN lines |
|  | TGCAGATTCGACACTGGAAG |  |
| qZFN-F | TGAATGGTGGAAGGTGTATCC | Expression analysis of ZFN in rice |
| qZFN-R | AAGCTGTGCTTTGTAGTTACCCTTA |  |
| qI-SceIF | GCTGTCTCCTCCTCACAAG | Expression analysis of I-SceI in rice |
| qI-SceIR | GGGTCAGGTAGTTCTCCACC |  |
| qUbi-F | CGCAAGTACAACCAGGACAA | Reference gene for expression analysis in rice |
| qUbi-R | GCTGTGACCACACTTCTTCTT |  |
| qPDS-F | GCAGAGGAATGGGTTGGAC | Reference gene for expression analysis in rice |
| qPDS-R | GTGAACCTTGCCGACCTCT |  |
