# Supplementary material for: Utility of I-SceI and CCR5-ZFN nucleases in excising selectable marker genes from transgenic plants
Source: BMC Res Notes. 2019 May 14;12:272. doi: 10.1186/s13104-019-4304-2 (PMC6518718; doi:10.1186/s13104-019-4304-2)
Supplement: Supplementary file 2 — Additional file 2: Figure S1. Molecular analysis of rice lines transformed with ZFN overexpression construct. (a) ZFN overexpression construct containing maize Ubiquitin-1 (ZmUbi) promoter, ZFN coding region and nopaline synthase (nos) 3’ transcription terminator. Primer positions and their product size are shown. (b) PCR analysis of 13 primary transgenic plants (T0) representing 11 transgenic events. (c) PCR analysis of T1 progeny from three T0 plants # 1, 2-1 and 3. d, e PCR analysis of additional T1 progeny from line #3. Product sizes are shown. Arrows indicate expected products in each gel. The PCR conditions for Figures (b–d) are mentioned in the main text. The PCR for 0.09 kb product (Figure e) was performed at 95 °C for 3 min followed by 30 cycles of 95 °C for 30 s, 60 °C for 30 s, and 72 °C for 30 s. [file 13104_2019_4304_MOESM2_ESM.pptx]

## Slide 1
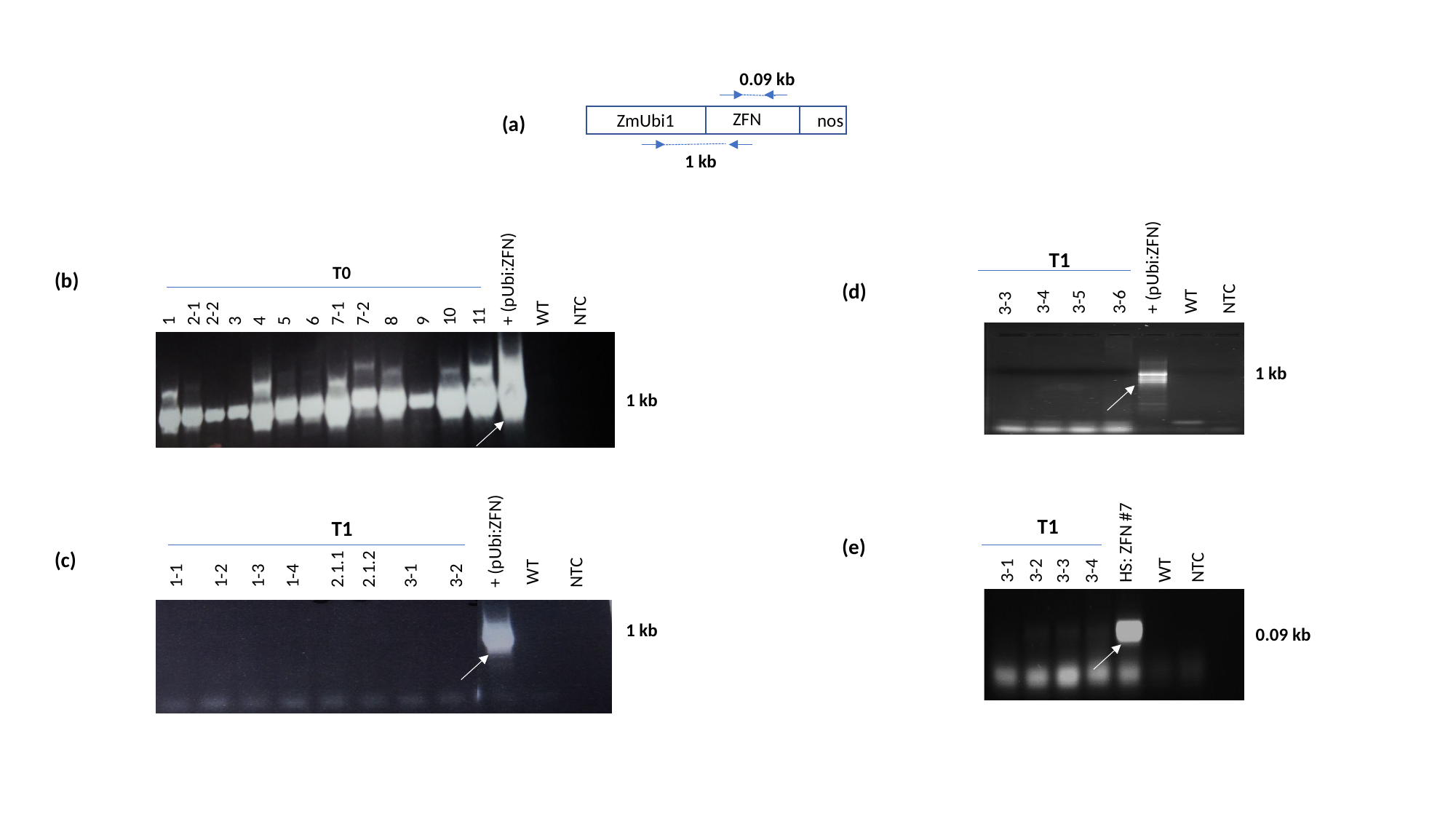

0.09 kb
ZFN
ZmUbi1
nos
1 kb
(a)
T1
+ (pUbi:ZFN)
3-5
3-6
3-4
NTC
3-3
WT
T0
+ (pUbi:ZFN)
WT
NTC
2-1
2-2
3
4
5
6
7-1
7-2
8
9
10
11
1
(b)
(d)
1 kb
1 kb
+ (pUbi:ZFN)
NTC
T1
2.1.1
2.1.2
1-1
1-2
1-3
1-4
3-1
3-2
T1
HS: ZFN #7
WT
NTC
3-3
3-4
3-2
3-1
0.09 kb
WT
(e)
(c)
1 kb
